# *MetaTopics*: an integration tool to analyze microbial community profile by topic model

**DOI:** 10.1186/s12864-016-3257-2

**Published:** 2017-01-25

**Authors:** Jifang Yan, Guohui Chuai, Tao Qi, Fangyang Shao, Chi Zhou, Chenyu Zhu, Jing Yang, Yifei Yu, Cong Shi, Ning Kang, Yuan He, Qi Liu

**Affiliations:** 1 Department of Central Laboratory, Shanghai Tenth People’s Hospital, School of Life Sciences and Technology, Tongji University, Shanghai, China; 2 Department of oral medicine, Shanghai Engineering Research Center of Tooth Restoration and Regeneration, School of Stomatology, Tongji University, Shanghai, China; 3 School of Life Science and Technology, Huazhong University of Science and Technology, Wuhan, China

**Keywords:** Metagenomics, R, Topic model, Microbial community, Disease status

## Abstract

**Background:**

Deciphering taxonomical structures based on high dimensional sequencing data is still challenging in metagenomics study. Moreover, the common workflow processed in this field fails to identify microbial communities and their effect on a specific disease status. Even the relationships and interactions between different bacteria in a microbial community keep unknown.

**Results:**

*MetaTopics* can efficiently extract the latent microbial communities which reflect the intrinsic relations or interactions among several major microbes. Furthermore, a quantitative measurement, *Quetelet Index*, is defined to estimate the influence of a latent sub-community on a certain disease status for given samples. An analysis of our in-house oral metagenomics data and public gut microbe data was presented to demonstrate the application and usefulness of *MetaTopics*. To preset a user-friendly R package, we have built a dedicated website, https://github.com/bm2-lab/MetaTopics, which includes free downloads, detailed tutorials and illustration examples.

**Conclusions:**

*MetaTopics is* the first interactive R package to integrate the state-of-arts topic model derived from statistical learning community to analyze and visualize the metagenomics taxonomy data.

**Electronic supplementary material:**

The online version of this article (doi:10.1186/s12864-016-3257-2) contains supplementary material, which is available to authorized users.

## Background

High-throughput sequencing techniques have been extensively applied in microbial metagenomics to study microbe diversity and community profiles from mixed DNA samples. Designing computational models to investigate the microbial community profile is a key step to recognize the microbial functions related to their host samples [1].

A common scenario in metagenomics study is to cluster or classify multiple samples represented by their OTU profiles based on 16S rRNA pyrosequencing. However, normal unsupervised clustering or supervised classification only provide the subdivisions of the samples, but fail to decipher the latent microbial community structures, their interactions as well as their correlation to specific disease status of such samples. Here, the latent microbial community or the sub-community, is represented by a group of bacteria, where their interactions are biologically or pathologically related to specific environment or disease status etc. To this end, we presented the first R package *MetaTopics*, which addresses the following issues: (1) how to identify microbial communities and their functions related to a specific disease status and (2) what relationships and interactions exist between different bacteria in a microbial community.

*MetaTopics* is developed to infer the microbial community structure across multiple samples based on a powerful statistical learning model, i.e. the topic model, originally derived from text community mining [2]. The topic model is a computational framework which was originally designed to uncover the hidden thematic structure in document collections [2, 3]. The basic idea of this model assumes that each topic consists of highly correlated words and each document contains several different topics with a certain probability distribution, and the distribution of such potential topics can be inferred by a set of given documents together with their word frequency representations. In particular, a Bayesian based method called Latent Dirichlet Allocation (LDA) can be used in such inference [4]. There are limited applications of the topic model in biological areas [5–9], and it is proven to achieve robust performance with tolerance to common noise of samples, which greatly exists in OTU assignment in metagenomics study [6]. So using the topic model to analyze metagenomics data could be an available way to decipher microbial community profiles.

By using the topic model, *MetaTopics* is developed to address the aforementioned questions we have raised by inferring the potential microbial community and bacteria interaction with both clustering and classification of the samples, and identifying the influence of a latent sub-community on a certain disease status.

## Methods and implementation

### Topic model for metagenomics study

Topic model, a type of statistical model, is originally used in machine learning and natural language processing area for latent “topics” discovery in a particular set of documents [1]. The basic idea of this model is that it assumes that each topic consists of the highly correlated words and each document may contain several different topics with a certain probability distribution, and the distribution of such potential topics can be inferred by given the set of documents together with their word frequency representations. In particularly, the Bayesian based model *Latent Dirichlet Allocation* [2] can be used in such inference. In the application of this model for text processing, each document follows a probability distribution over topics, and each topic follows a probability distribution over words. This generative hierarchical model, assumes that a word in a document is generated through two steps, i.e., a topic in a document is chosen with a certain probability, and then a word in the topic is chosen with a certain probability. The generative process of topic model is formulated as follows: *θ*_*d*_ and ∅ _*t*_ are respectively the distribution over topics of document *d* and that over words of topic *t*.

$$ {\uptheta}_d\sim Dirichlet\left(\alpha \right) $$

$$ {\varnothing}_t\sim Dirichlet\left(\beta \right) $$

Here *α* and *β* are hyper parameters following Dirichlet distributions. For generating word *i* in document *d*, topic *Z*_*d*,*i*_ is first sampled from document’s distribution over topics, and then word *W*_*d*,*i*_ is sampled from topic’s distribution over words based on the following distributions,

$$ {\mathrm{Z}}_{d,i}\Big|{\uptheta}_d\sim Multinomial\left({\uptheta}_d\right) $$

$$ {\mathrm{W}}_{d,i}\Big|{\mathrm{Z}}_{d,i},{\varnothing}_{{\mathrm{Z}}_{d,i}}\sim Multinomial\left({\mathrm{Z}}_{d,i}\right) $$

In this study, the topic model is utilized to process our metagenomics data. We made a perfect analogy between text mining and microbial community detection, where documents can be analogized to the samples in metagenomics study and the words frequency in a document can be analogized to the OTUs abundance for a given sample. We formed a joint probability of bacteria taxa to each sample by integrating parameter *θ* into *φ* and applied collapsed Gibbs sampling to assign the bacteria taxa of each sample to topics. Detailed information can be referred to [2].

### R package *MetaTopics* implementation

**Fig. 1:**
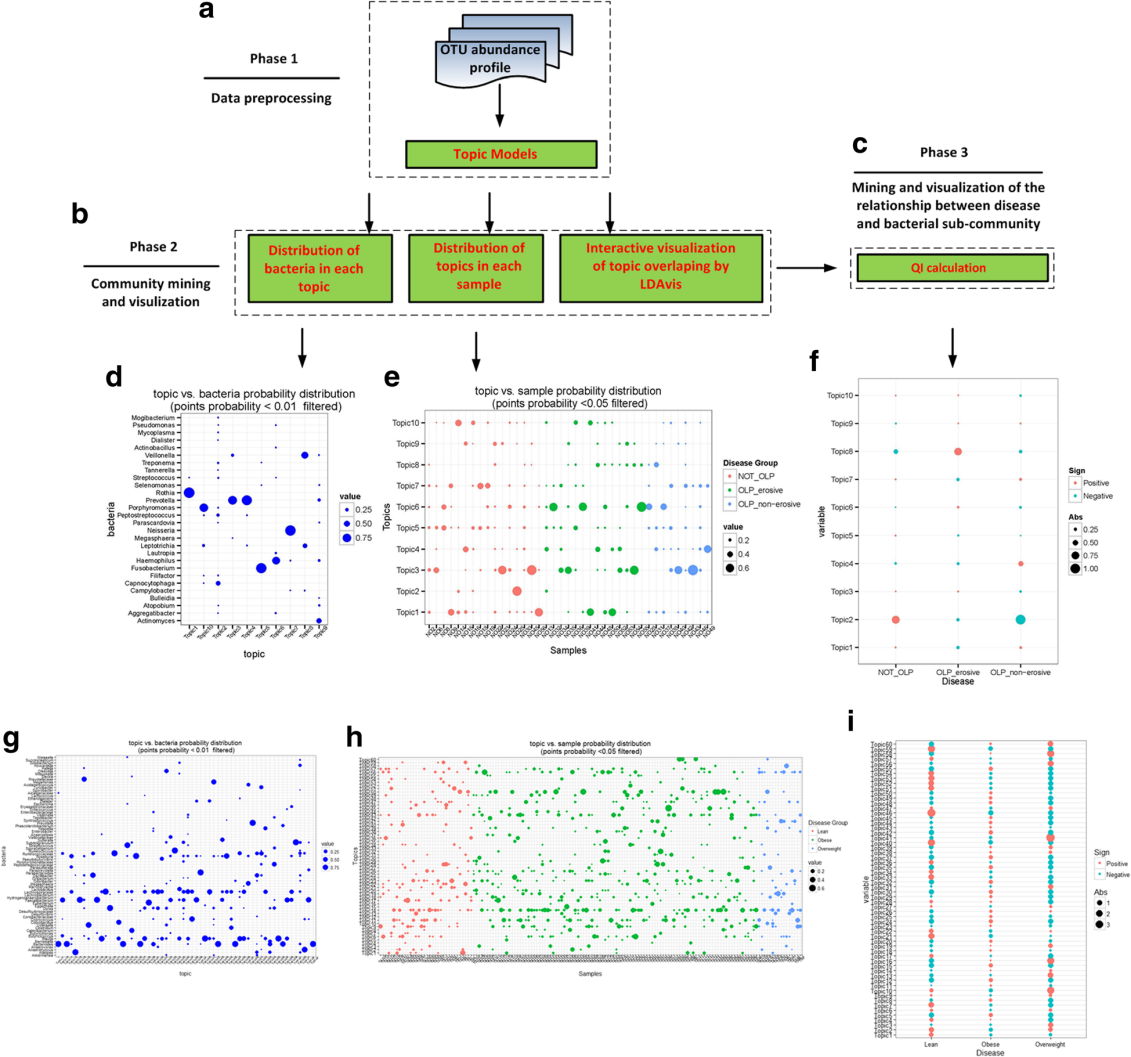
A computational pipeline of MetaTopics in the analysis of metagenomics samples. Panels **a**-**c** indicate the sequential steps for MetaTopics to mine the metagenomics data. Panels **d**-**f** and **g**-**i** indicate the mining results on our in-house oral samples and public gut samples respectively

*MetaTopics* is an R package, designed purposely to support the workflow of applying topic model to metagenomics data, with the following sample analysis and visualization functions (Fig. 1). Several functions are built to visualize the abundance and diversity of the microbial profiles over the individual samples. The core topic model used in *MetaTopics* is integrated from the R package *topicmodels* [10], which provides LDA models and Correlated Topic Models (CTM) [2] (Fig. 1a). Each topic, viewed as a microbe sub-community, biologically representing a group of high correlated bacteria functioning similarly in a disease status, can be interpreted by the probability distribution and the profile of bacteria. And each sample can be represented by these sub-communities with different degree. Various interactive visualization approaches based on ggplot2 [11] and LDAvis [12] are incorporated to show the composition of each sub-community and each sample for comparison. After identifying the dominant microbes in each sub-community, these sub-communities can be visualized by the level of overlap to indicate the community interaction, which guides the deep investigation of the microbe interactions (Fig. 1b). In addition, considering the substantial needs in the analysis of the relationship between each sub-community and a certain disease status, the *Quetelet Index* (*QI*) [13] is defined to estimate the relative change of the observation frequency of a specific latent sub-community among all the samples compared to that among the samples with a certain disease status (Fig. 1c). *QI* quantitatively describes the degree of the influence of a specific topic on a certain disease (see Additional file 1, Defining *QI* for topic and disease status relationship analysis Section, for more details).

## Results and discussion

### Data descriptions and preprocessing

As an example, *MetaTopics* was firstly applied on the in-house oral metagenomics dataset which contains 39 oral human samples. 23 of these samples are patients with two subtypes of oral lichen planus (OLP, 9 OLP_non-erosive and 14 OLP_erosive) and 16 of them are controls. There are totally 129 bacteria OTUs in genus level counted from these samples. In addition, a public gut microbe 16S RNA sequencing dataset [14] was used to test the efficiency of *MetaTopics*. The dataset includes 154 human faecal samples classified by the corresponding individual BMI category (104 obese, 16 overweight and 34 lean). There are totally 190 bacteria OTUs in genus level counted from these samples, revised by NCBI taxonomy database. Before applying *MetaTopics*, the bacteria which exist in very few samples as well as the samples with very few bacteria taxons were filtered. The package *BiotypeR* which is developed for the gut enterotype analysis [15] was used to remove genera with low abundance across all samples to decrease the noise. The term-frequency inverse document frequency (tf-idf) score [2] was used in *MetaTopics* to select the “document vocabulary”, i.e. bacteria taxon here. Finally, 88 and 176 genera were retained for these two datasets respectively for the further analysis.

### Results analysis

The number of topics for the given samples was determined in a data-driven way [10]. Perplexity and likelihood were used in *MetaTopics* for topic number identification [10]. By using 5-fold cross-validation, 10 topics in oral dataset and 60 in gut dataset were determined using LDA algorithm coupled with Gibbs Sampling in *MetaTopics* [4, 10].

As a result, one matrix that consists of bacteria occurring probability distribution in each topic was visualized in Fig. 1d and g separately for two datasets (points with probability no more than 0.01 are not shown). Another matrix representing the microbial composition of each sample over topics was visualized in Fig. 1e and h separately for two datasets (points with probability no more than 0.05 are not shown). Additional file 1: Figures S1 and S2 separately integrate all the topics in a multidimensional scaling way to represent the topic interactions over two datasets.

As a quantitative measure to describe the degree of the influence of a specific topic on a certain disease, *QI* was calculated for all the 10 topics (Fig. 1f) of oral dataset and 60 topics (Fig. 1i) of gut dataset. As a result, the community detection, visualization and *QI* calculation by *MetaTopics* (Fig. 1) do provide us the biological insight of the given samples over two different datasets. The topics identified by *MetaTopics* represent the biological sub-community bacteria group that may be related to specific disease status. In the oral dataset it shows that topic 5 is very common in these samples. And topic 8 mainly consists of *Veillonella* and *Leptotrichia*, seems specified in OLP_erosive group. In another independent experimental validation, *Leptotrichia* is proven to activate basal keratinocytes and antigen-presenting cells in OLP (data not shown). Such findings further indicate that bacteria interaction rather than single bacteria might also be served as one of the causative factors of OLP, where bacterial infection may influence the immuno-pathogenetic process of this disease [16]. In the gut dataset, Lachnospiraceae, Blautia and Faecalibacterium from Firmicutes phylum and Bacteroides from Bacteroidetes phylum are very common in these samples. Topic 1, mainly composed of bacteria from Bacteroidetes phylum, has a clear decrease in obese group compared to the lean one. Topic 16, mainly composed of bacteria from Actinobacteria phylum, has a clear increase in obese group compared to the lean one. These findings are consistent with Turnbaugh’s study [14]. The multidimensional scaling of topics shows these topics roughly cluster into two groups, Firmicutes/Actinobacteria and Bacteroidetes phylum. Further biological meanings of the topics identified by *MetaTopics* are waited to be explored by the microbiologic scientist.

## Conclusion

*MetaTopics* provides a powerful platform by incorporating topic models into metagenomics data analysis, to discover and visualize the microbial community and the relationships between bacteria and diseases with impressive insights.

## Availability and requirements

**Project name:** *MetaTopics*

**Project home page:** https://github.com/bm2-lab/MetaTopics

**Operating system(s):** Linux, Mac and PC

**Programming language:** R

**Other requirements:** dplyr, ggplot2, reshape, topicmodels, LDAvis, slam, BiotypeR

**License:** GPL (> = 2)

**Any restrictions to use by non-academics:** No

## Additional file

Additional file 1: Supplementary methods, figures and tables. (DOCX 799 kb)
