# Supplementary material for: MetaTopics: an integration tool to analyze microbial community profile by topic model
Source: BMC Genomics. 2017 Jan 25;18(Suppl 1):962. doi: 10.1186/s12864-016-3257-2 (PMC5310276; doi:10.1186/s12864-016-3257-2)
Supplement: Additional file 1: — Supplementary methods, figures and tables. (DOCX 799 kb) [file 12864_2016_3257_MOESM1_ESM.docx]

Additional file 1

**1.** **Defining *Quetelet Index* (*QI*) for topic and disease status relationship analysis**

*Quetelet Index* (*QI*) is an index introduced by Mirkin in 2001[3] to represent the correlation between variables *k* and *l* as the relative change in the probability of *l* when *k* is taken into account.

QI(*l/k*) = (P(*l/k*) - P(*l*)) / P(*l*)

In *MetaTopics*, considering the substantial needs in the analysis of the relationship between each sub-community and a certain disease status, the original definition of *QI* is adopted to estimate the relative change of the observation frequency of a specific latent sub-community among all the samples compared to that among the samples with a certain disease status. In this way, *QI* is defined to quantitatively describe the degree of the influence of a specific topic on a certain disease. Specifically, in order to analyze the correlation between the established latent sub-communities and disease status, it is presumed that a sub-community will be identified as a meaningful observation in an individual sample if it has an occurring probability bigger that a threshold, for example, 0.05. Then the *QI* is applied as the formula below:

QI (Topic *i* | Disease *j*) = (P(Topic *i* | Disease *j*) - P(Topic *i* )) / P(Topic *i*)

Where P (Topic *i*) is estimated by the occurring frequency of the meaningful observed topic *i* among all the samples, while P(Topic *i* | Disease *j* ) is estimated by the occurring frequency of the meaningful observed topic *i* among the samples with a certain disease status *j .* As an example, , the *QI* for each latent sub-community under three different status (NOT_OLP, OLP_no-erosive, and OLP_erosive for our oral metagenomics data; lean, obese, and overweight for the gut metagenomics data ) are separately shown in Table 1 and 2. A positive value of *QI* indicates that the considered topic occurs more frequently than average in a certain status, while a negative one indicates the topic occurs less frequently than average.

| **Table S1. Calculation of *QI*s for the 10 topics under 3 different** **status for our or** |
| --- |
| **our oral metagenomics data** |

|  | **NOT_OLP** | **OLP_no-erosive** | **OLP_erosive** |
| --- | --- | --- | --- |
| Topic 1 | 0.08 | 0.15 | -0.23 |
| Topic 2 | 0.77 | -1 | -0.23 |
| Topic 3 | 0.03 | -0.14 | 0.07 |
| Topic 4 | -0.13 | 0.46 | -0.19 |
| Topic 5 | 0.08 | -0.01 | -0.1 |
| Topic 6 | 0 | -0.17 | 0.14 |
| Topic 7 | 0.06 | 0.19 | -0.23 |
| Topic 8 | -0.41 | -0.26 | 0.74 |
| Topic 9 | -0.05 | 0.06 | 0.03 |
| Topic 10 | 0.05 | -0.12 | 0.03 |

| **Table S2. Calculation of *QI*s for the 60 topics under 3 different status for the or** |
| --- |
| **gut metagenomics data** |

|  | **lean** | **obese** | **overweight** |
| --- | --- | --- | --- |
| Topic1 | 0.850758 | -0.24805 | -0.30982 |
| Topic2 | 0.962925 | -0.45313 | 0.756818 |
| Topic3 | -0.13631 | -0.09767 | 0.9325 |
| Topic4 | 0.439479 | 0.002594 | -1 |
| Topic5 | -1 | 0.503891 | -1 |
| Topic6 | 0.143115 | -0.11536 | 0.420956 |
| Topic7 | 1.159218 | -0.24805 | -1 |
| Topic8 | -0.38308 | 0.289049 | -1 |
| Topic9 | 0.110455 | -0.09767 | 0.380357 |
| Topic10 | 0.439479 | -0.4987 | 2.220833 |
| Topic11 | -0.00344 | 0.041155 | -0.25673 |
| Topic12 | -1 | 0.503891 | -1 |
| Topic13 | -0.4602 | -0.06007 | 1.415625 |
| Topic14 | -0.09764 | 0.010076 | 0.153731 |
| Topic15 | -1 | 0.503891 | -1 |
| Topic16 | -1 | 0.002594 | 2.220833 |
| Topic17 | 0.591003 | -0.05017 | -1 |
| Topic18 | -0.21483 | 0.093739 | -0.12159 |
| Topic19 | -0.38308 | 0.002594 | 0.840476 |
| Topic20 | -0.42421 | 0.203113 | -0.35583 |
| Topic21 | 1.591061 | -0.54883 | -0.03375 |
| Topic22 | 0.818289 | -0.28763 | 0.017105 |
| Topic23 | -0.05534 | 0.080922 | -0.39609 |
| Topic24 | -1 | 0.503891 | -1 |
| Topic25 | -0.42421 | 0.203113 | -0.35583 |
| Topic26 | -0.53731 | 0.181629 | 0.035268 |
| Topic27 | 0.079609 | -0.06007 | 0.207813 |
| Topic28 | 0.328749 | 0.041155 | -1 |
| Topic29 | -0.13631 | 0.203113 | -1 |
| Topic30 | -1 | 0.503891 | -1 |
| Topic31 | -0.13631 | -0.09767 | 0.9325 |
| Topic32 | -1 | 0.503891 | -1 |
| Topic33 | 1.159218 | -0.24805 | -1 |
| Topic34 | 0.660937 | -0.19021 | -0.25673 |
| Topic35 | -1 | 0.503891 | -1 |
| Topic36 | 0.850758 | -0.14063 | -1 |
| Topic37 | -1 | 0.503891 | -1 |
| Topic38 | -0.38308 | 0.074208 | 0.380357 |
| Topic39 | -0.64013 | 0.127918 | 0.610417 |
| Topic40 | 2.238827 | -0.62403 | -1 |
| Topic41 | -0.56816 | -0.39844 | 3.83125 |
| Topic42 | -1 | 0.503891 | -1 |
| Topic43 | -0.21483 | 0.093739 | -0.12159 |
| Topic44 | -0.38308 | 0.289049 | -1 |
| Topic45 | -0.28026 | 0.253243 | -1 |
| Topic46 | 3.318436 | -1 | -1 |
| Topic47 | 0.259544 | -0.18539 | 0.610417 |
| Topic48 | -0.4602 | 0.315905 | -1 |
| Topic49 | -0.42421 | 0.303372 | -1 |
| Topic50 | -0.43673 | 0.176958 | -0.15978 |
| Topic51 | 1.159218 | -0.24805 | -1 |
| Topic52 | 1.159218 | -0.24805 | -1 |
| Topic53 | 1.591061 | -0.39844 | -1 |
| Topic54 | 1.159218 | -0.24805 | -1 |
| Topic55 | -1 | 0.503891 | -1 |
| Topic56 | -0.36494 | -0.11536 | 1.557721 |
| Topic57 | 0.439479 | -0.06902 | -0.53988 |
| Topic58 | -1 | 0.002594 | 2.220833 |
| Topic59 | 2.454749 | -0.69922 | -1 |
| Topic60 | -1 | 0.127918 | 1.415625 |

**2.** **Interactive visualization of topic model by *LDAvis***

*MetaTopics* seamlessly incorporates package *LDAvis*, which is presented by Carson and Kenneth to offer a web-based tool for interactive visualization of Topic Models [1-5]. The MDS (Multidimensional Scaling) method combined with the marginal distributional of each topic is used to show the relationship among topics. Besides, two parameters, *saliency* and *relevance*, are used to rank the importance of the words, i.e. the bacteria in this application, overall and in specific topic respectively. *Saliency*, introduced by Chuang et. al [6], is used to quantitatively measure the overall importance of the bacteria for visualization. *Relevance* balances the conditional probability P(bacteria|topic), i.e. the topic-specific probability, and the lift probability, defined as P(bacteria|topic)/P(bacteria) with a weight [4]. A snapshot of the interactive visualization of the analysis results on our oral metagenomics data and gut metagenomics data are separately shown in Figure 1 and 2.


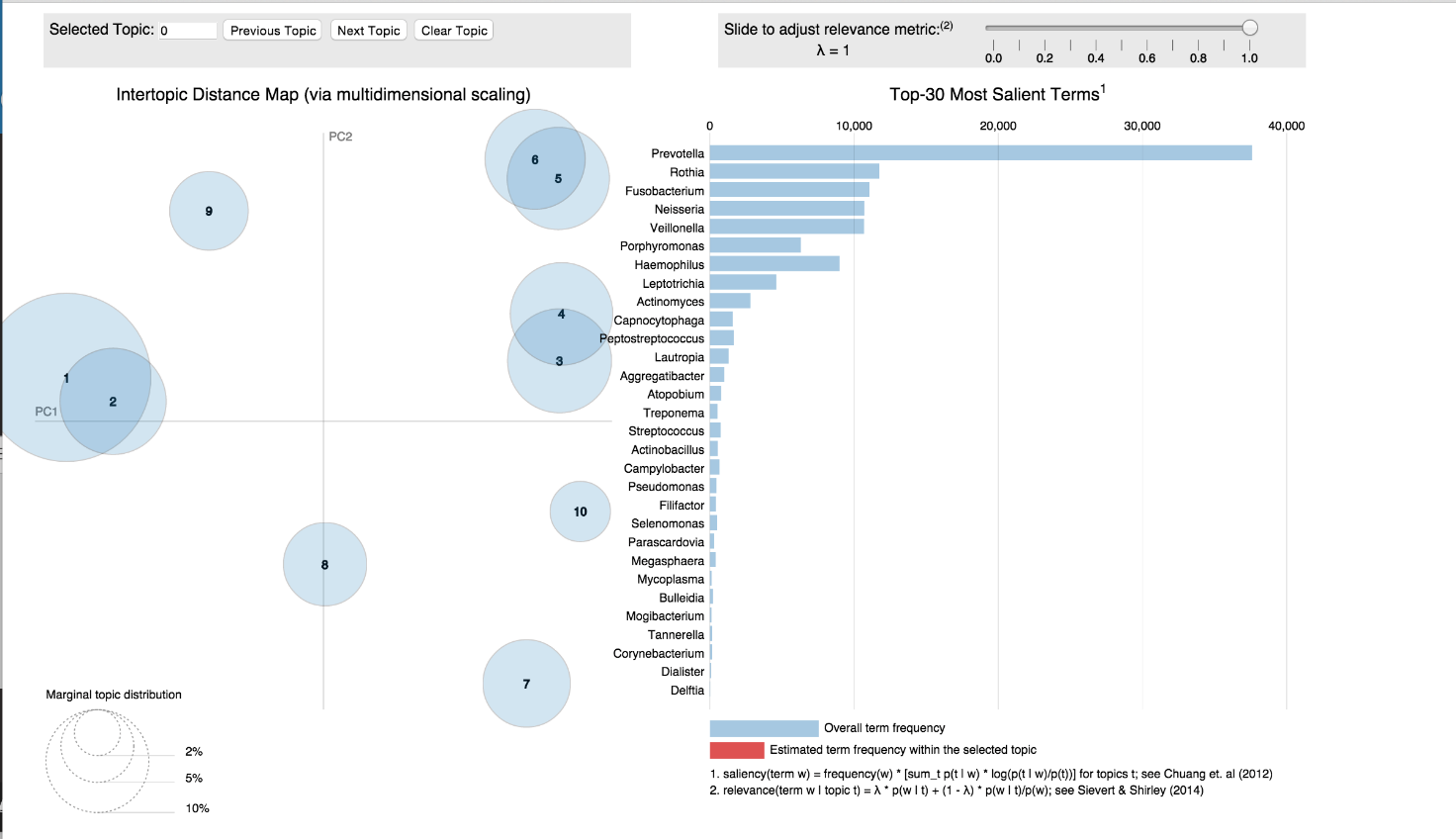


**Figure S1. A snapshot of the interactive visualization of the analysis results on our oral metagenomics data**

**
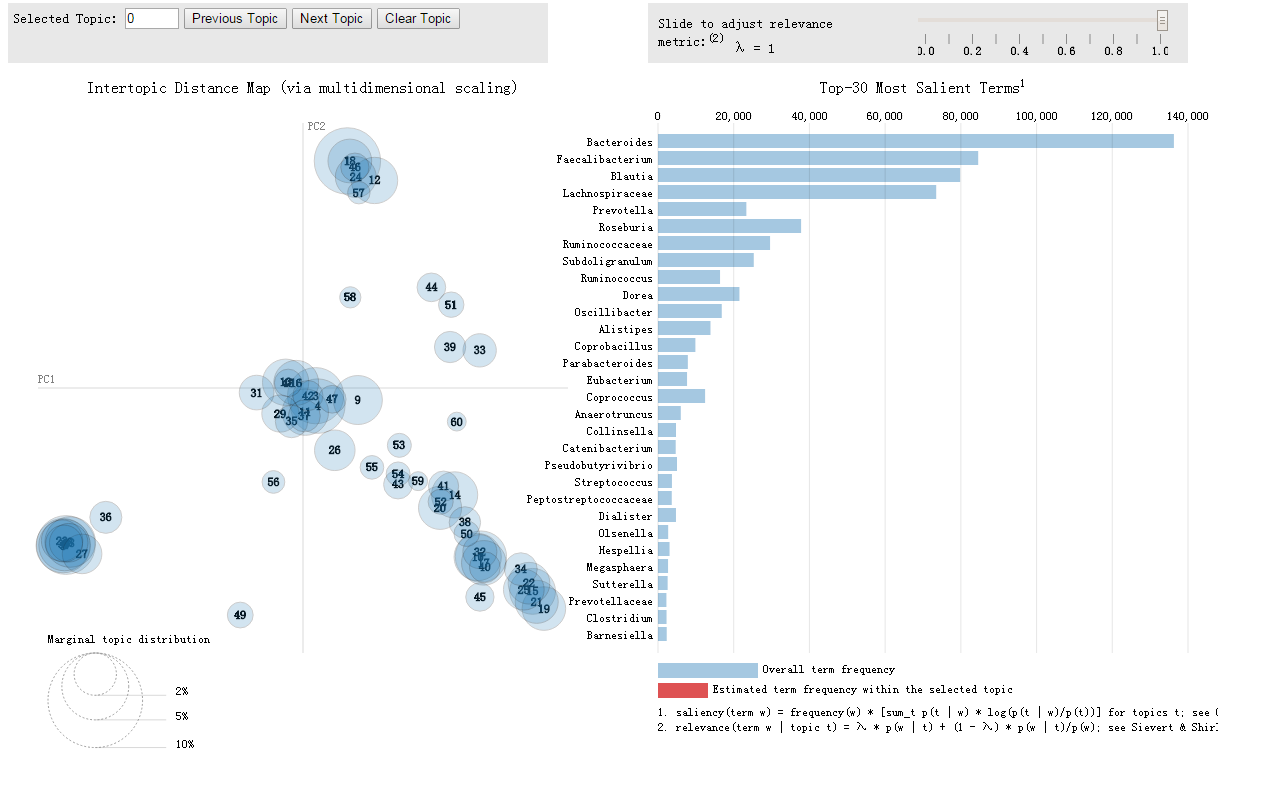
**

**Figure S2. A snapshot of the interactive visualization of the analysis results on public gut metagenomics data**

**Reference**

[1] Blei, D.M. Probabilistic topic models. *Communications of the ACM* 2012;55(4):77-84.

[2] Blei, D.M., Ng, A.Y. and Jordan, M.I. Latent dirichlet allocation. *the Journal of machine Learning research* 2003;3:993-1022.

[3] Mirkin, B. Eleven ways to look at the chi-squared coefficient for contingency tables. *The American Statistician* 2001;55(2):111-120.

[4] Carson Sievert and Kenny Shirley, LDAvis: Interactive Visualization of Topic Models. R package version 0.2. <http://CRAN.R-project.org/package=LDAvis>, 2015.

[5] Carson Sievert and Kenny Shirley, LDAvis: A method for visualizing and interpreting topics, *Proceedings of the Workshop on Interactive Language Learning, Visualization, and Interfaces*, pages 63–70,2014.

[6] Jason Chuang, Christopher D. Manning and Jeffrey Heer, Termite: Visualization Techniques for Assessing Textual Topic Models. *AVI*, 2012.
